# Plant Origin Regulates the Response of *Solidago canadensis* Reproductive Traits to Long-Term Warming and Nitrogen Addition

**DOI:** 10.3390/plants14111711

**Published:** 2025-06-04

**Authors:** Xiaohui Zhou, Xin Chen, Xin Luo, Yanling Wu, Juanjuan Li, Jianxin Ren, Jingji Li

**Affiliations:** 1 State Key Laboratory of Geohazard Prevention and Geoenvironment Protection, Chengdu University of Technology, Chengdu 610059, China; xiaohuizhou@cdut.edu.cn (X.Z.); xinchen202103@163.com (X.C.); 18227401382@163.com (X.L.); wuyanling0312@163.com (Y.W.); 18582714889@163.com (J.L.); 13808016883@163.com (J.R.); 2 Key Laboratory of Synergetic Control and Joint Remediation for Soil & Water Pollution, Ministry of Ecology and Environment, Chengdu University of Technology, Chengdu 610059, China; 3 College of Ecology and Environment, Chengdu University of Technology, Chengdu 610059, China

**Keywords:** climate warming, plant invasion, provenance, reproduction, *Solidago canadensis*

## Abstract

Climate warming and nitrogen (N) deposition have already occurred and will continue to occur, profoundly affecting exotic plant invasion. Most studies on the effects of climate change focus on plant growth, biomass, and leaf traits, with limited reports on reproductive responses. We selected *Solidago canadensis* from North America and China as focal species and conducted a long-term common garden experiment simulating climate warming and N deposition to examine how climate warming, N addition, and plant origin influence its reproductive traits. Chinese *Solidago canadensis* exhibited significantly greater ramet height, more robust ramet diameters, longer and wider inflorescences, and higher seed mass compared to North American *Solidago canadensis*. Long-term warming and plant origin alone or in combination significantly influenced reproductive traits, while N addition did not influence these traits. The vegetative propagation of a native population was sensitive to warming and N addition, while the generative propagation of an invasive population was sensitive to their combined effects. These findings suggest that the reproductive strategies of *Solidago canadensis* varied with their origin, and plant origin might be important in mediating climate change effects on their reproduction under plant invasion.

## 1. Introduction

Plant invasion constitutes a severe threat to ecosystems and regional economic development, emerging as one of the most critical global challenges [1,2,3]. When exotic plants reach new regions, they encounter a variety of environmental factor changes; in response, these invasive plants can undergo adaptive evolution more rapidly through alterations in their growth characteristics [3,4,5,6]. Consequently, climate change has been identified as one of the key drivers of plant invasion [3,4,7]. Climate warming and increased nitrogen (N) deposition, as significant indicators of climate change, profoundly impact the reproductive development of exotic invasive plants [6,7].

Temperature, a core component of climate, strongly influences plant conditions, profoundly altering physiological, growth, defense, and reproductive processes [6,7,8,9]. It is predicted that the global temperature will increase by approximately 2 °C over the next 50 years, a critical threshold for eliciting ecosystem feedback [10,11]. Under climate warming, invasive plants exhibit significant genetic variation, leading to enhanced growth, reproductive capacities, and increased invasive potential, which gives them an advantage in the context of climate change [12]. For example, longer warm periods may also enable invasive plants to produce fruit and reproduce within the growing season in areas with climates which, in the past, would not have supported such rapid growth [4].

Nitrogen is the most commonly limiting element for plant growth [13], and the availability of resources in habitats is a key factor determining community susceptibility to exotic plant invasion [14]. Global atmospheric N deposition ranges from 0.05 to 2 g N m^−2^ yr^−1^ due to intensified industrial and agricultural activities, with annual N deposition in southern of China at approximately 4 g∙m^−2^∙yr^−1^ [15,16,17]. Moreover, N deposition is expected to continue increasing in the future [15,16,17]. Consistent research has indicated that N fertilizer promotes plant growth and reproduction, increases nitrogen compound content, and decreases carbon compound content, redirecting more carbon resources towards growth [18,19].

Another key factor influencing invasion success is the plant population origin [6,20]. Invasive plants exhibit adaptive responses to new selective pressures, leading to phenotypic shifts in growth patterns and resource allocation in the invaded habitat [21]. In addition, the interaction between genetic origins and climate may also affect their invasion potential [22]; for example, native populations of *Solidago canadensis* are more sensitive to nitrogen than those from invaded ranges [20]. Reproductive traits are crucial for the successful establishment, spread, and invasion success of species in novel habitats [23]. Climate change may alter reproductive traits, particularly in relation to trade-off dynamics [24].

*Solidago canadensis*, a perennial herb native to North America, was introduced to Shanghai, China, in the 1930s as an ornamental plant, and it has become one of the most destructive exotic invasive species, rapidly spreading to the western and southern regions of China [20,25,26,27]. *Solidago canadensis* is capable of both asexual reproduction, through the production of tiller shoots from rhizomes, and sexual reproduction, via the production of numerous small seeds [20,25]. Previous studies have shown that *S. canadensis* is highly sensitive to climate warming and N deposition [28], which may enhance its ability to acquire resources and increase its invasiveness [29]. Research has also shown that warming limits the biomass and relative competitiveness of *S. canadensis*, whereas N deposition enhances them [30]. However, most studies focus on plant growth, biomass, and leaf traits, with limited reports on reproductive responses.

Studying the reproductive responses of invasive plants to warming and N deposition is crucial for predicting their invasiveness. To address this, we selected *S. canadensis* from North America (SNA, native origin) and China (SCN, invasive origin) as our focal species and conducted a common garden experiment simulating climate warming and N deposition to examine how climate warming, N addition, and plant origin influence its reproductive traits. Specifically, we tested the following two hypotheses: (1) warming and N addition could influence the reproduction of *S. canadensis* depending on origin and (2) the three experimental factors (warming, N addition, and origin) exhibited differential influences on the associations among reproductive traits.

## 2. Results

### 2.1. Reproductive Traits Under Warming, N Addition, and Plant Origin

**Figure 1:**
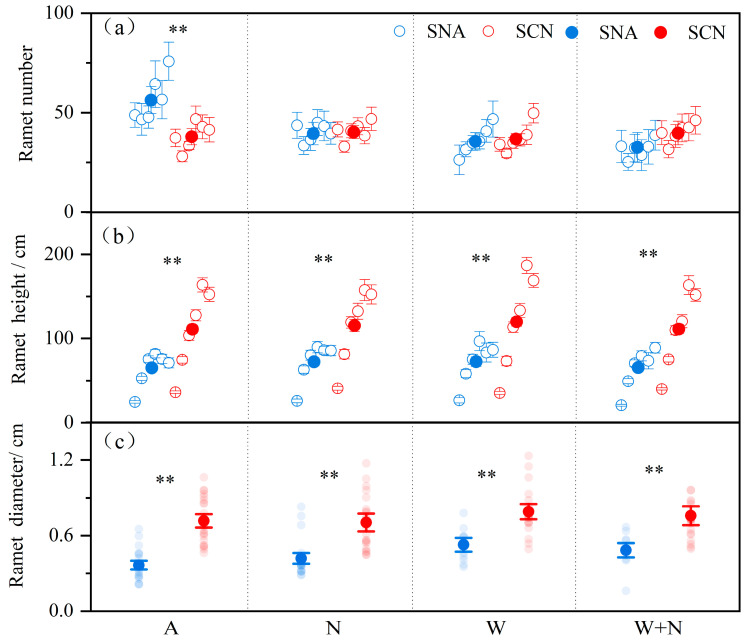
The effects of warming, nitrogen addition, and origin on the ramet numbers (**a**), height (**b**), and diameter (**c**) of *S. canadensis*. Note: SNA: *S. canadensis* from North America; SCN: *S. canadensis* from China; A: ambient; N: N addition; W: warming; W + N: warming plus N addition. In a and b, the hollow points represent the mean of each measurement ± SE for SCN and SNA, while the solid points indicate the average of measurements ± SE (n = 6). In c, transparent points are observed values, and solid points represent the mean ± SE (n = 18). ** *p* < 0.01 indicate significant differences in reproductive traits between SNA and SCN.

Warming significantly affected the ramet number and ramet diameter of *S. canadensis* (Table 1: *p* < 0.05) and did not significantly affect the ramet height (Table 1). The ramet height and diameter of SCN (invasive origin) were greater than those of SNA (native origin) regardless of the environmental treatment (Table 1: *p* < 0.05; Figure 1), and the ramet number was also significantly affected by the interaction between warming and origin (Table 1: *p* < 0.05). Warming reduced the ramet number of SNA, whereas it did not affect the ramet number of SCN (Figure 1). Nitrogen addition had no significant effect on the tillering characteristics of *S. canadensis* (Table 1). Furthermore, the interaction effects among warming, N addition, and origin did not significantly affect the ramet number, ramet height, and ramet diameter (Table 1).

**Figure 2:**
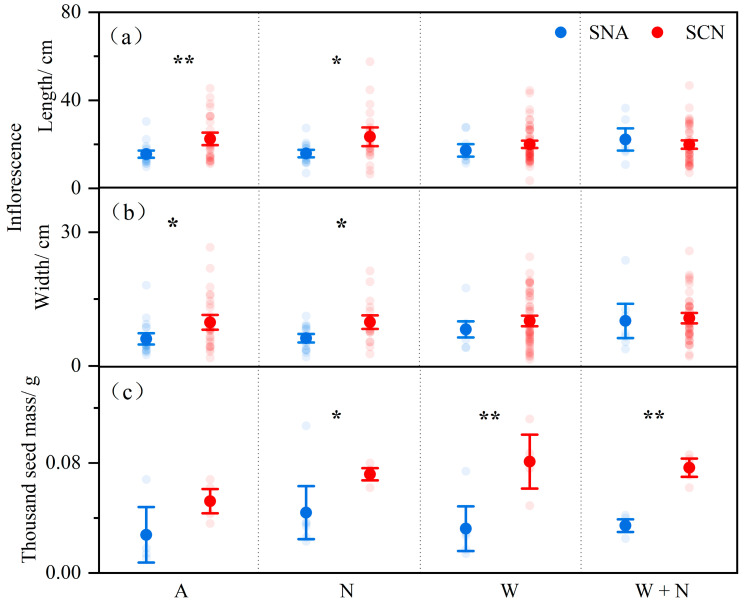
The effects of warming, nitrogen addition, and origin on the inflorescence length (**a**), width (**b**), and thousand seed mass (**c**) of *S. canadensis*. Note: Transparent points are observed values and solid points represent the mean ± SE (n = 60 for inflorescence length and width; n = 5 for thousand seed mass). SNA: *S. canadensis* from North America; SCN: *S. canadensis* from China; A: ambient; N: N addition; W: warming; W + N: warming plus N addition. * *p* < 0.05 and ** *p <* 0.01 indicate significant differences in reproductive traits between SNA and SCN.

There were significant differences in inflorescence length and thousand seed mass between SCN and SNA (Table 2: *p* < 0.05). Warming significantly affected the inflorescence width (Table 2: *p* < 0.05) and did not significantly affect the thousand seed mass of *S. canadensis* (Table 2). The inflorescence length was also significantly affected by the interaction between warming and origin (Table 2: *p* < 0.05); the inflorescence length of SCN was longer than that of SNA under the control, but this difference was not observed under warming (Figure 2). Nitrogen addition did not significantly affect the sexual reproduction traits of *S. canadensis* (Table 2). The interaction effects among warming, N addition, and origin did not significantly affect the inflorescence length, inflorescence width, and thousand seed mass of *S. canadensis* (Table 2).

### 2.2. Relative Change in Reproductive Traits Under Different Environments

**Figure 3:**
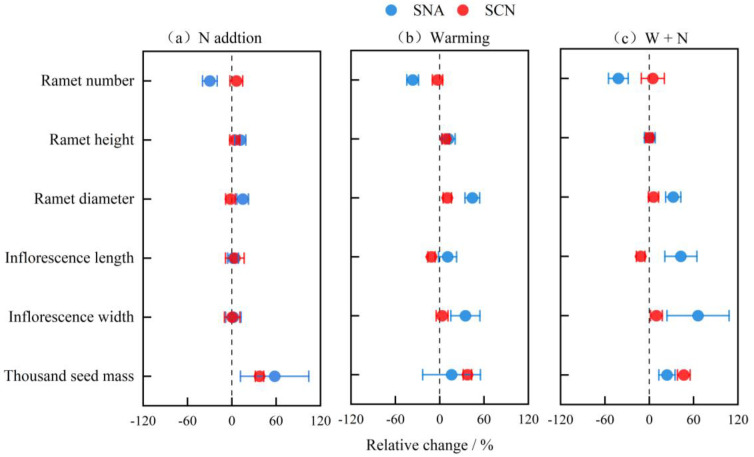
The relative changes in reproductive traits of *S. canadensis* from SNA and SCN under warming, N addition, and their combination. Note: The data in the graph represent the mean ± SE (n = 6 for ramet number and ramet height; n = 6 for ramet diameter; n = 6 for inflorescence length and width; n = 5 for thousand seed mass). SNA: *S. canadensis* from North America; SCN: *S. canadensis* from China; W + N: warming plus N addition.

When N was added, the relative change in the ramet number of SNA was significantly decreased compared to the control, and both SNA and SCN showed elevated relative changes in thousand seed mass related to the control; only the SCN exhibited a significant increase (Table 3: *p* < 0.05; Figure 3a). Warming led to a significant reduction in the relative change in the ramet number and an increase in the relative change in the ramet diameter of SNA compared to the control (Table 3: *p* < 0.05; Figure 3b). The relative change in the inflorescence length of SCN under warming treatment was significantly less compared to the control (Table 3: *p* < 0.05; Figure 3b). Under warming and N addition, the relative change in the ramet diameter of SNA was significantly greater than that in the control (Table 3: *p* < 0.05; Figure 3c). In contrast, for SCN, the relative change in the inflorescence length was reduced, while the relative change in the thousand seed mass was higher compared to the control (Table 3: *p* < 0.05; Figure 3c).

### 2.3. Potential Associations Among Reproductive Traits

**Figure 4:**
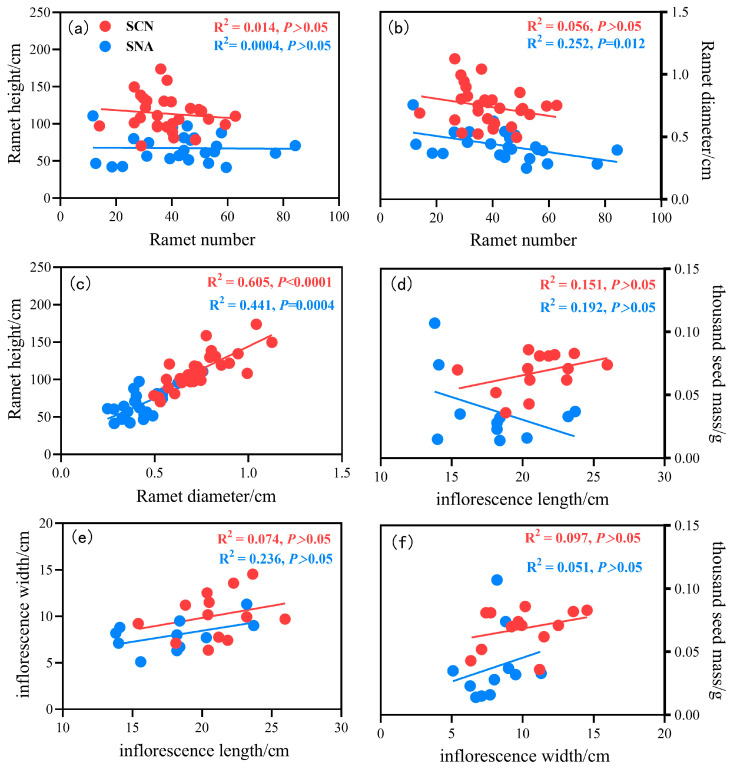
Linear regression relationship of reproductive traits of *S. canadensis* from different origins. (**a**) the linear regression relationship between ramet height and ramet number, (**b**) the linear regression relationship between ramet diameter and ramet number, (**c**) the linear regression relationship between ramet height and ramet diameter, (**d**) the linear regression relationship between thousand seed mass and inflorescence length, (**e**) the linear regression relationship between inflorescence width and inflorescence length, and (**f**) the linear regression relationship between thousand seed mass and inflorescence width. Note: SNA: *S. canadensis* from North America; SCN: *S. canadensis* from China The blue solid points (n = 11–24) and blue solid line represent SNA, while the red solid points (n = 14–29) and red solid line represent SCN.

The linear regression results were as follows: all reproductive trait relationships were consistent between SNA and SCN, except for that between the thousand seed mass and the inflorescence length; the thousand seed mass was negatively associated with the inflorescence length in SNA, whereas a positive association was observed in SCN (Figure 4). In addition, the ramet diameter significantly decreased with the increase in the ramet number in SNA, while the relationship was not significant in SCN (Figure 4b). The ramet height significantly increased with the increase in ramet diameter both in SNA and SCN (Figure 4c). The interaction between the ramet height and ramet number, as well as the interactions among inflorescence length, inflorescence width, and thousand seed mass, exhibited similar patterns and were not significant in SNA and SCN (Figure 4a,e,f).

**Figure 5:**
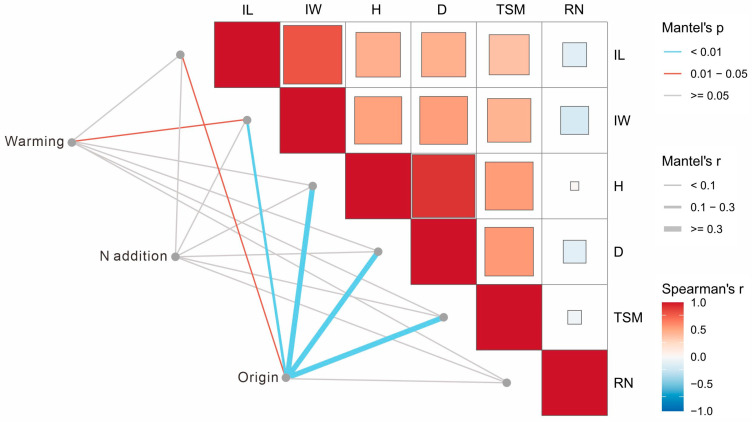
The Mantel test for the associations of warming, N addition, and plant origin with reproductive traits of *S. canadensis*. The line color indicates the Mantel test *p*-value of the association between environmental factors and reproductive traits, the brown line represents 0.01 < *p* < 0.05, the blue line represents *p* < 0.01, and the gray line represents *p* ≥ 0.05. The line width corresponds to the Mantel test r value. The color of the box represents pairwise correlations between reproductive traits, with deeper color saturation denoting stronger correlation. Note: IL: inflorescence length; IW: inflorescence width; H: ramet height; D: ramet diameter; TSM: thousand seed mass; RN: ramet number.

We used the Mantel test to identify the plant reproductive factors that were shaped by warming, N addition, and plant origin (Figure 5). Climate warming was significantly correlated with inflorescence width (IW); N addition was not significantly correlated with any of the reproductive traits; plant origin was significantly correlated with inflorescence length (IL), inflorescence width (IW), ramet height (H), ramet diameter (D), and thousand seed mass (TSM) (Figure 5: *p* < 0.01).

**Figure 6:**
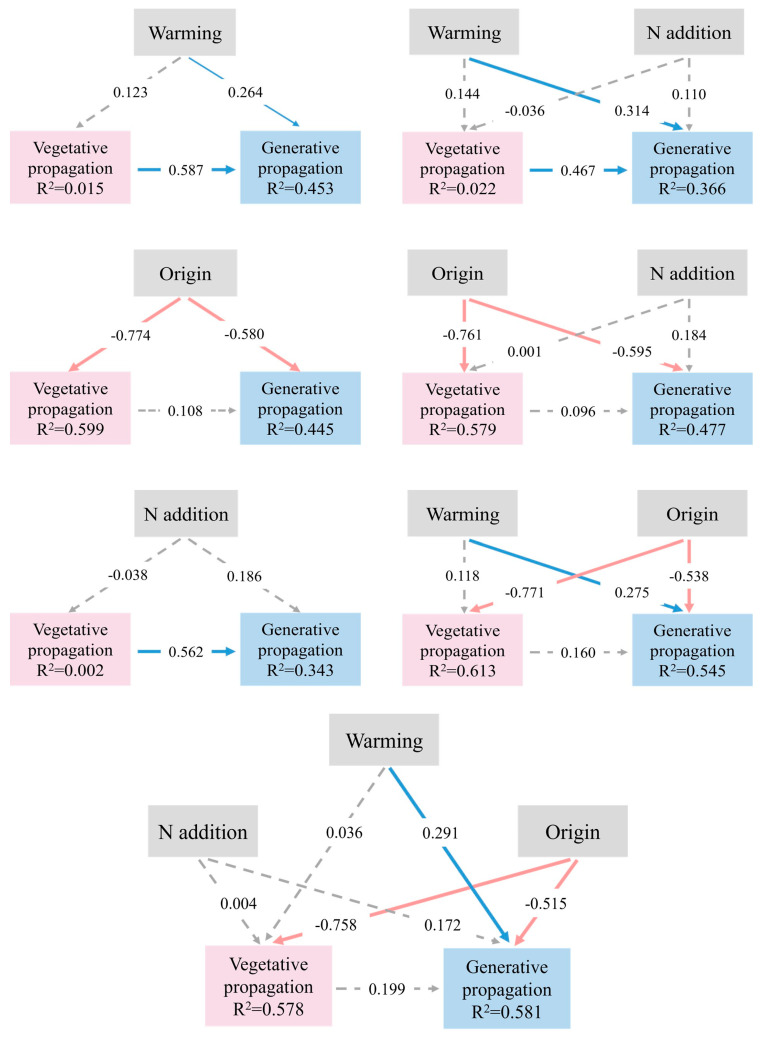
Pathway models examining the impacts of climate warming, N addition, or/and plant origin on vegetative and generative propagation. The red solid arrow indicates a significant negative association between explanatory and response variables, the blue solid arrow indicates a significant positive association between explanatory and response variables, and the gray dashed arrow indicates a nonsignificant association between explanatory and response variables. The width of a solid arrow indicates the intensity of associations; the numbers associated with pathways represent standard path coefficients.

The SEM analysis revealed the effects of warming and plant origin on vegetative and generative propagation (Figure 6). Warming had a significant direct positive effect on generative propagation but no significant effect on vegetative propagation; SNA had lower characteristic values of both vegetative propagation and generative propagation than SCN; and vegetative propagation and generative propagation had positive and significant correlations under warming, N addition, or interaction between warming and N addition (Figure 6).

## 3. Discussion

Based on the long-term warming and N addition experiment, we explored the effects of climate warming and N addition on the vegetative and generative reproductive traits of *S. canadensis*. Two key findings of this study were as follows: (1) warming and plant origin alone or in combination significantly influenced reproductive traits, while N addition did not influence these traits; (2) plant origin regulated the sensitivity of reproductive traits to warming and N addition. Thus, this study provides insights into the effects of climate change on plant reproduction and potential invasion risks. Plant geographical origin is crucial for predicting the effects of ongoing climate change on plant distributions and invasions.

Provenance experiments often reflect the genetic responses of plants to different ecological environments [31]. Invasive plants are typically larger in their introduced range than in their native range [32]. Our results revealed significant differences in five reproductive traits (ramet height, ramet diameter, inflorescence length, inflorescence width, and thousand seed mass) of *S. canadensis* between the two population origins (Figure 2 and Figure 3). Overall, this difference between native and invasive origins could be attributed to the climate of origin, with native *S. canadensis* experiencing a cool climate and invasive *S. canadensis* encountering a warm climate [33,34,35].

Specifically, *S. canadensis* from invasive origins exhibited higher reproductive superiority compared to those from native populations. This superiority manifested primarily in the following two key aspects: (1) *Solidago canadensis* from the invasive range exhibited greater ramet height and more robust ramet diameters than those from the native range (Figure 1). This finding is consistent with studies on *Triadica sebifera*, which revealed that invasive populations could capture more sunlight, thereby gaining a significant competitive advantage [36]. The result also implies that *S. canadensis* may have developed enhanced growth traits, facilitating rapid population dominance, thus overshadowing native plants and augmenting its competitiveness in invaded habitats [37]. (2) *Solidago canadensis* from the invasive range had longer and wider inflorescences and higher seed mass than those from the native range (Figure 2). Hautier et al. [38] indicated that larger plant individuals allocated more resources to sexual reproduction. Consistent with this, *S. canadensis* from invasive populations are larger than those from native populations, with their inflorescences bearing more seeds and higher seed quality [20,35]. Previous studies have shown that *S. canadensis* from invasive populations have larger seeds, faster germination, and higher germination rates than those from native populations [6,35]. The biogeographical difference may be attributed to rapid evolution and slow adaptation [39]. For example, climate change is expected to drive rapid evolutionary responses, potentially reshaping integrated sets of life history traits; migration or gene flow from warmer regions may help reduce slow adaptation if those populations are better suited to current conditions than local ones [39]. The results indicate that *S. canadensis* from invasive origins may allocate energy towards producing more high-quality seeds, providing an advantage for long-distance dispersal and successful invasion [40]. We also found that the ramet number of *S. canadensis* from both population origins exhibited no significant variance (Figure 1), which contrasts with the initial stages of the experiment conducted in 2012–2013 [41] but is consistent with findings from studies conducted in 2014–2015 [34]. It indicates that ramet numbers might be contingent upon resource availability. Taken together, there are potential genetic differences between the origins, and their performance may also indicate physiological adaptation to the invaded habitat.

Growth–defense trade-offs are common in invasive plants, but there are some exceptions [42]. We found that the ramet height significantly increased with ramet diameter regardless of the population origin (Figure 4 and Figure 5). In this study, we selected stem diameter as a proxy to assess defensive investment in *S. canadensis*. The stem plays a crucial role in providing mechanical support and facilitating water absorption in plants, with its thickness directly affecting the ability of plants to resist external disturbances; meanwhile, greater stem thickness at a given height indicates stronger resistance to physical stress and herbivory, suggesting a higher resource allocation to defense during plant growth and competition [34,41]. Plant height represents an indicator of growth potential [20]. Our results indicate that *S. canadensis* exhibits strong growth and defense capabilities, which is consistent with findings on invasive *Centaurea* species [43]. The coordinated increase in growth and defense could enhance the success of invasion.

Climatic warming could influence plant growth, population dynamics, flowering phenology, and reproductive success [44]. Our study found that long-term warming significantly reduced the ramet number of *S. canadensis* and increased its diameter and inflorescence width (Figure 1, Figure 2 and Figure 5). Plants can facilitate resource transfer among species through clonal integration, promoting their growth in adverse environments [45]. However, the response of *S. canadensis* reproductive traits to warming remains uncertain. For example, Ren et al. [30] found that two years of warming inhibited the diameter and plant height of *S. canadensis*, whereas Peng et al. [34] revealed that four years of warming increased the ramet number but decreased the plant height. The discrepancies might be related to the duration of warming, as long-term warming depleted underground carbohydrate and nutrient reserves, leading to a more pronounced impact on reproductive effort and success compared to short-term effects [46,47].

The growth and reproductive strategies of plant populations exhibit diverse responses to climate warming. Increasing evidence indicates that the environment experienced by plant populations significantly influences their responses to surrounding conditions [48]. The effects of warming on the ramet number and inflorescence length were dependent on population origins (Figure 1 and Figure 2). The ramet number reflects reproductive strength in competitive environments and dispersal potential. Warming decreased the ramet number in native populations but had no significant effect on invasive populations (Figure 1). This suggests that prolonged warming does not significantly affect the clonal reproduction of *S. canadensis* from invasive populations. Inflorescences serve as containers for seed nutrient accumulation, and their morphology directly affects seed production [49]. Warming led to a reduction in the inflorescence length of *S. canadensis* from invasive populations (Figure 2). This suggests that warming might cause invasive *S. canadensis* to allocate resources for improving seed quality. It was worth noting that N addition had no significant impact on the six reproductive characteristics of *S. canadensis* (Figure 1, Figure 2 and Figure 5). This may be due to the varying reproductive strategies of plant species in response to N deposition or the long-term cumulation of N deposition (10 years), which might have reached a threshold that *S. canadensis* could effectively utilize.

We also found that four of the six reproductive traits showed significant variance in response to warming and N addition; the vegetative propagation of the native population was sensitive to warming and N addition, and the generative propagation of the invasive population was responsive to their combined effects (Figure 3). The trade-off between sexual and clonal reproduction is shaped by both the plant intrinsic characteristics and environmental factors [50]. Compared to clonal reproduction, sexual reproduction generates offspring with genetic diversity, thereby enhancing their capacity to adapt to heterogeneous changing environments and promoting dispersal across longer distances [51]. Invasive plants from different provenances exhibit variation in their growth and reproductive strategies [52]. For instance, *Lythrum salicaria* from its native range tended to exhibit an earlier flowering period and a preference for sexual reproduction, whereas individuals from invasive regions typically exhibited an extended growth period, aimed at increasing plant height, and had a preference for clonal reproduction [53]. Overall, these differences in sensitivity might be attributed to genetic variations, selective pressures, and the phenomenon of rapid evolution [54,55,56].

## 4. Materials and Methods

### 4.1. Experimental Location and Materials

The experiment was established at the Chengdu University of Technology in Chengdu City, Sichuan Province, China (30.68° N, 104.14° E, elevation 512 m). This site is located in a subtropical humid monsoon climate zone, with an annual average temperature of 16.1 °C and annual precipitation of 918.2 mm. In this experiment, we selected *S. canadensis* from both native (North America, *S. canadensis* from North America, SNA) and invasive (China, *S. canadensis* from China, SCN) origins. The seeds of North American *S. canadensis* were collected from five different sites in Montana, USA, and the seeds of the Chinese *S. canadensis* were collected from five different sites in Ningbo, Zhejiang [35]. Flow cytometry was employed to determine the ploidy levels of the seeds, revealing that *S. canadensis* plants from both China and the United States possess the same ploidy level [34].

### 4.2. Long-Term Warming and N Addition Experiment

**Figure 7:**
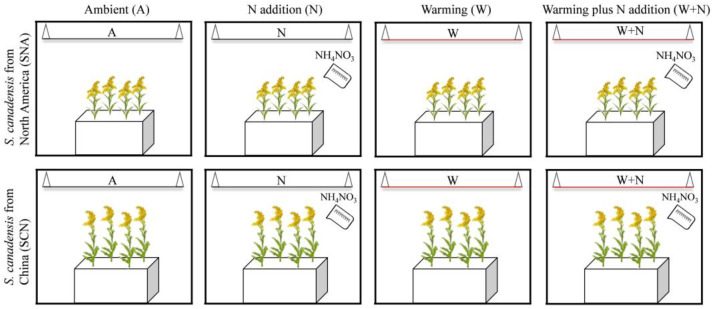
The experimental design involving three factors (Factor 1: origin; Factor 2: temperature; Factor 3: N addition). The plant origins are *S. canadensis* from North America (SNA) and *S. canadensis* from China (SCN). The four climate scenarios are the ambient (A), climate warming (W), N addition (N), and climate warming plus N addition environment (W + N).

To test the effects of climate warming, N deposition, and plant origin on the growth of *S. canadensis*, we conducted a long-term warming experiment in June 2012. The experiment employed a three-factor factorial design, included two plant origins (North America and China), two temperatures (ambient and warming), and two N additions (not included and included), thereby forming eight distinct treatment combinations, with each combination replicated eight times, yielding a total of 64 experimental pots (Figure 7).

We prepared 64 experimental pots (30 cm length × 30 cm width × 20 cm depth), each filled with a 1:1 mixture of sand and loam. The sand component was obtained from fine sand collected along the riverbank, while the loam was sourced from the experimental site itself. These components were sieved through a 1 cm aperture mesh, thoroughly mixed, and then placed into pots [20,34]. The plant seeds of *S. canadensis* were collected from five distinct populations in North America and China, respectively. For the warming and N addition experiment, seeds from each origin were pooled and sown randomly. We planted 20 seeds in each pot, and after two months of growth, four individual plants remained per pot, with a uniform spacing of 10 cm between each plant and from the edge of the pot. When *S. canadensis* had grown for two months, the experimental warming treatment was initiated using MSR-2420 infrared radiators (Kalglo Electronics, Bethlehem, PA, USA). The heater was positioned 1.5–2.0 m above the soil surface to simulate a climate warming scenario of 2 °C [34]. For the unwarmed treatment, a “dummy” heater, identical in shape and size to the infrared radiator, was suspended to replicate the shading effect of an infrared radiator (Figure 7). To simulate atmospheric N deposition, ammonium nitrate (NH_4_NO_3_, 99.5% purity) was applied at an annual rate of 4 g N∙m^−2^ year^−1^, corresponding to 1.028 g per pot [34]. The initial nitrogen application was carried out in August 2012. Subsequently, N was added in a wet pulse in March, April, May, and June each year. Meanwhile, an equal volume of water was supplied to the mesocosms without N addition. The warming and N addition treatments have been ongoing since 2012, encompassing a decade of continuous observation. A detailed description of the long-term experimental design has been given previously [6,34,35].

In the experiment, we measured six reproductive traits of *S. canadensis* from July to December in 2022. We selected the ramet number, ramet height, and ramet diameter as indicators of vegetative propagation, as well as inflorescence length, inflorescence width, and thousand seed mass as indicators of generative propagation. We recorded the ramet number, ramet height, and ramet diameter of *S. canadensis* at 45-day intervals from July to November in 2022. The ramet number was determined by counting the number of tillers in each experimental pot. We randomly selected three ramets from each pot to measure height using a ruler, defined as the distance from the leaf tip to the soil surface. We measured the ramet diameter by using a caliper to record the diameter at the base of the ramets. We measured the inflorescence length, inflorescence width, and thousand seed mass of *S. canadensis* in December 2022. We randomly selected ten plants from each pot to measure the length and width from the inflorescence lowest point to the top. It was observed that the ramet number of *S. canadensis* per pot had increased from the initial four to several dozen due to the 10-year duration of the experiment. To determine seed mass, we randomly selected 100 seeds from each combination, weighed their air-dried mass, and this assessment was repeated five times. Due to the extremely small seed of S. canadensis, the 100 seed weight was used instead of the standard thousand seed weight. Consequently, the mass of a thousand seeds was calculated as ten times the mass of a hundred seeds.

Expanded on in the above determinations, we calculated the relative change in each of the six reproductive traits to quantify the effect of N addition, warming, or warming plus N addition on each trait of the native and invasive populations from *S. canadensis*. The relative change is (Tm − Ta)/Ta × 100%, where Tm and Ta are the values of a given trait in the native or invasive population grown under manipulative (N addition, warming, or warming plus N addition) and ambient conditions, respectively.

### 4.3. Data Analyses

All data processing and statistical analyses were performed using R 4.0.2 [57], while graphical representations were created using Origin 2021. The normality of data and homogeneity of variance were tested using the Shapiro-Wilk test and Bartlett’s test, respectively. The inflorescence length and width were log-transformed to ensure variance homogeneity. We used a three-factor analysis of variance (ANOVA) to test the effects of warming, N addition, and plant origin on the ramet number, ramet height, ramet diameter, inflorescence length, inflorescence width, and thousand seed mass of *S. canadensis*. The differences in ramet number, ramet height, ramet diameter, inflorescence length, inflorescence width, and thousand seed mass between SNA and SCN under a given environment were tested using the “TukeyHSD function”. We used independent sample *t*-tests to analyze the relative changes in reproduction traits, assessing whether these changes were equivalent to zero.

To examine differences in reproductive trait relationships between SNA and SCN, we used linear regression to analyze the pairwise relationships between the ramet number, ramet height, ramet diameter, inflorescence length, inflorescence width, and thousand seed mass. Partial Mantel tests were used to quantify the correlations of explanatory variables (i.e., ramet number, ramet height, ramet diameter, inflorescence length) with climate warming, N addition, and origin.

We also used structural equation models (SEMs) to explore the associations between vegetative propagation and generative propagation under climate warming, N addition, and plant origin. In partial Mantel tests and SEMs, binary values (0 = SCN, 1 = SNA) were used to assess relationships between plant origin and reproductive traits. Vegetative propagation encompassed the ramet number, ramet height, and ramet diameter; generative propagation encompassed inflorescence length, inflorescence width, and thousand seed mass. We employed the partial least squares method in SmartPLS 4.1.0.3 software to establish SEMs and utilized 5000-sample size bootstrapping to test the significance of path coefficients [58]. The reliability and validity of SEMs were confirmed using Cronbach’s alpha (Alpha, Alpha > 0.7), composite reliability (CR, CR > 0.8), and average variance extracted (AVE, AVE > 0.5) [59].

## 5. Conclusions

This study might help us to understand the vegetative and generative propagation of invasive plants under rapid environmental changes. This study provides some implications. For example, long-term warming and plant origin alone or in combination significantly influenced reproductive traits. *Solidago canadensis* from invasive populations exhibited significantly greater ramet height, more robust ramet diameters, longer and wider inflorescences, and higher seed mass compared to that from native populations. Long-term warming reduced the ramet number in native populations but had no effect in invasive populations and mitigated the difference in inflorescence length between native and invasive populations. Ramet height increased significantly with ramet diameter across both population origins. The vegetative propagation of the native population was sensitive to warming and N addition, and the generative propagation of the invasive population was responsive to their combined effects. However, we failed to record plant phenology because it plays a crucial role in driving plant invasion and is strongly driven by climate change [20]. Our study also did not evaluate the genetic variation between SNA and SCN. Genetic variation between populations, especially those from different geographic regions, plays a crucial role in shaping plant fitness, adaptation, and ecological performance. For a comprehensive analysis of trait differences between the two origins, a large number of seed populations would need to be sampled if we are to adequately assess differences between populations and their ecological significance and adaptability in diverse habitats.

## Figures and Tables

**Table 1:** The effects of warming (W), nitrogen (N) addition, and origin on the ramet number, height, and diameter of *S. canadensis*. Note: * indicates significant difference at *p* < 0.05; ** indicates significant difference at *p* < 0.01.

	Ramet Number		Ramet Height		Ramet Diameter	
	*F*	*p*	*F*	*p*	*F*	*p*
Warming (W)	4.461	0.040 *	1.084	0.299	9.747	0.002 **
N addition (N)	0.883	0.352	0.000	0.996	0.092	0.762
Origin (O)	1.585	0.214	144.941	<0.001 **	106.523	<0.001 **
W × N	0.527	0.471	3.266	0.073	1.063	0.304
W × O	4.454	0.040 *	0.384	0.537	0.759	0.385
N × O	3.382	0.072	0.597	0.441	0.243	0.623
W × N × O	0.359	0.552	0.169	0.681	0.462	0.498

**Table 2:** The effects of warming (W), nitrogen (N) addition, and origin on the inflorescence length, width, and thousand seed mass of *S. canadensis*. Note: * indicates significant difference at *p* < 0.05; ** indicates highly significant difference at *p* < 0.01.

	Inflorescence Length		Inflorescence Width		1000 Seed Weight	
	*F*	*p*	*F*	*p*	*F*	*p*
Warming (W)	0.378	0.540	4.696	0.031 *	1.174	0.287
N addition (N)	0.620	0.432	0.897	0.345	1.607	0.214
Origin (O)	4.516	0.035 *	7.759	0.006 **	29.491	<0.001 **
W × N	0.577	0.449	0.096	0.757	2.058	0.161
W × O	4.807	0.030 *	2.920	0.089	2.134	0.154
N × O	0.831	0.363	0.002	0.963	0.014	0.908
W × N × O	0.621	0.432	0.004	0.952	0.147	0.704

**Table 3:** Results from the *t*-test for the relative changes in reproductive traits of *S. canadensis* from SNA and SCN under warming, nitrogen addition, and their combination. Note: * indicates significant difference at *p <* 0.05; ** indicates highly significant difference at *p <* 0.01.

	N Addition (N)		Warming (W)		W + N	
	SNA	SCN	SNA	SCN	SNA	SCN
Ramet number	0.021 *	0.501	0.019 *	0.695	0.051	0.767
Ramet height	0.140	0.569	0.214	0.120	0.917	0.992
Ramet diameter	0.072	0.792	0.001 **	0.081	0.009 **	0.420
Inflorescence length	0.818	0.748	0.406	0.030 *	0.097	0.048 *
Inflorescence width	0.844	0.964	0.111	0.684	0.167	0.234
Thousand seed mass	0.266	0.003 **	0.702	0.115	0.094	0.005 **

## Data Availability

Data is contained within the article.

## Abbreviations

The following abbreviations were used in this manuscript:

SNA	*Solidago canadensis* from North America
SCN	*Solidago canadensis* from China
A	Ambient
W	Warming
N	Nitrogen
W + N	Warming plus N addition
O	Origin
